# Understanding the Role of Users’ Psychological Needs on Relationship Quality in Short Video Applications

**DOI:** 10.3389/fpsyg.2022.858521

**Published:** 2022-04-21

**Authors:** Zhounan Huangfu, Lei Zhou, Jing Zhao, Sombat Kotchasit, Wanmei Chen

**Affiliations:** ^1^Design College, Zhoukou Normal University, Zhoukou, China; ^2^Valaya Alongkorn Rajabhat University, Pathum Thani, Thailand; ^3^Art Design College, Henan University of Engineering, Zhengzhou, China; ^4^Faculty of Humanities and Social Sciences, City University of Macau, Macau, Macau SAR, China; ^5^Henan Vocational University of Science and Technology, Zhoukou, China

**Keywords:** attachment theory, psychological needs, emotional attachment, relationship quality, attachment anxiety

## Abstract

Along with the rapid development of big data, artificial intelligence, and information technology, the relationship quality (RQ) between short video applications and users is important for the sustainable development of short video applications. However, the existing studies have explored the mechanism of the role of RQ in a limited way. In order to respond to this critical issue, this study constructs a theoretical model based on attachment theory and combined with self-determination theory, with autonomy needs (AN), competence needs (CN), and relationship needs (RN) as influencing factors, emotional attachment (EA) as mediating variables and relationship quality as outcome variables, and the moderating role of attachment anxiety (AA) in which this study also analyzes the mechanism of short video applications users’ psychological needs on relationship quality by combining the moderating role of AA. In this study, a sample of 512 university students using short video applications was used. The results of the data analysis indicated that EA was significantly influenced by psychological needs that played a positive role in relationship quality and mediated the relationship between psychological needs and relationship quality. The results of further analysis also revealed that attachment anxiety plays a moderating role in the relationship between emotional attachment and relationship quality. This study examines the intrinsic mechanism by which psychological needs affect relationship quality through emotional attachment, reveals the practical effects of short video applications users’ sustained use behavior, and provides a reference for innovative management and business practices of short video applications.

## Introduction

With the rapid development of big data, artificial intelligence, and information technology, short video applications has played a decisive role in the life of the public with its fissionable communication and ability to sink followers, and has become a topic of active participation and conversation among the public (Meng and Leung, 2021; Yang et al., 2021a). However, while short video applications have changed the way of life of the public, they also face the challenge of lack of sustained use. It has been suggested that users continue to use short video applications because they form emotional attachments (EAs) to short video applications, and that insight into the mechanisms that shape this attachment process is important for the sustainability of short video applications (Yang et al., 2021a,b). Obviously, the phenomenon of continuous use of local short video applications is already a popular phenomenon and has accounted for a significant proportion in the whole system of social virtual worlds (SVWs; Ma et al., 2021). In such a context, how local short video applications service providers can better operate their APP media, improve the quality of the relationship between users and short video applications, and enhance the conversion of users’ continuous use has become an urgent practical problem for short video applications developers to solve. Existing literature has discussed the relationship quality (RQ) between users and short video applications mainly from the following aspects.

First, with the advent of the information explosion era and the advancement of information technology, the number of platforms for the public to participate in leisure and entertainment is increasing, among which short video applications play the role of a platform for the relationship between operation, business, and marketing, whose main function is to create entertainment value and sharing value through the app platform where users can share their lives, meet more friends and learn about various interesting stories (Ahmed et al., 2019). In addition, the “anytime, anywhere” and “non-stop” nature of the APP platform also provides users with the convenience of communicating across space, increasing the social aspects of interactive communication and content distribution, and thus accessing more information as well as mass entertainment (Oghuma et al., 2016; Gan et al., 2018a). It also provides users with the convenience of communicating across space, increasing socialization in terms of interactive communication and content distribution, and thus access to more information as well as mass entertainment (Gan et al., 2018b). More and more consumers are now choosing to use local short video apps for mass entertainment (Gong et al., 2020). Especially in recent years, local mobile APPs that create and share content for users are popular with the majority of users, influenced by the rapid development of the mobile Internet and the widespread use of smartphones (Xie and Zhu, 2019). This shows that through short video applications platforms users are able to provide entertainment for others, as well as entertain themselves, and rely on marketing integration to create diverse business models and energize the platforms (Camacho et al., 2021). And with the increasing number of active users of local short video applications platforms at present, it is clear that short video applications has played an important role in the lives of the general public (Al-Debei et al., 2013; Chen et al., 2020). Therefore, the application value of short video applications platforms is further released in terms of user socialization and platform business realizations (Casalo and Romero, 2019).

Second, with the rising scale of local short video applications platform users, the commercial value of short video applications has received more attention. The commercialization of short video applications has attracted more companies to enter this field to compete for markets and users in order to reap the dividends of the technological revolution, and competition has led to the emergence of similar IT systems and equipment products in the market, which have sprung up in the lives of the public (Hayes et al., 2020). However, while the boom in short video applications has enriched the lives of the public, it has also intensified the level of commercialized competition (Mou et al., 2021). More seriously, the more prominent problem of short video applications homogeneity has in turn further intensified the fierce competition in short video applications (Zhao and Jie, 2020). In addition, the inherent low switching costs of short video applications platforms have led to a lack of loyalty among users, resulting in a shift between short video applications platforms. This indicates the importance of maintaining users to use the media platform again. In this regard, some foreign scholars point out that in the long run, the acceptance of the system depends on the continued adoption of the system by the users (Hsu et al., 2015; Hu and Zhang, 2016; Oghuma et al., 2016). Studies by domestic scholars also suggest that the long-term survival and ultimate success of information systems depend on users’ continuous usage behavior (Fan, 2017; Sun et al., 2020). Therefore, for local short video applications, users’ continuous use then becomes an important topic worthy of study nowadays (Li et al., 2021).

From the above analysis, it is clear that the issue of the relationship between users and short video applications is crucial. Although the research on short video applications platform users’ behavior has been conducted in a wide range of areas and there is a wealth of empirical studies, the previous literature has focused on consumers’ personal factors and system characteristics, but there is a lack of research on attachment as an important influencing factor driving consumers’ use of IT products or services. Attachment, as a construct based on relational processes, primarily characterizes the process of emotional relationships between an individual and the object of attachment (Yang et al., 2021a). RQ, on the other hand, is an assessment of the overall strength of the relationship and is an important outcome of relationship marketing (Guede and Filipe, 2019; Liquan and Yoon, 2021). There is a logical relationship of influence between this process and outcome. Previous research has addressed the effects of individual attachment on different dimensions of relationship quality, and study of Thomson (2006) found that individual attachment has a direct effect on relationship outcome variables such as satisfaction, trust, and commitment. However, the effect of EA on the quality of the relationship between users and short video applications in the context of short video applications use is an area that remains to be explored.

In order to respond to this critical issue, this study constructs a theoretical model with psychological needs [autonomy needs (AN), competence needs (CN), and relationship needs (RN)] as the influencing factors, emotional attachment as the mediating variable, attachment anxiety (AA) as the moderating effect, and relationship quality as the outcome variable, based on attachment theory and combined with self-determination theory, and takes the mass users of Tik Tok as the research object, and uses structural equation modeling (SEM) is used to analyze the mediating mechanism of relationship quality between users and short video applications and its boundary conditions.

## Theoretical Foundation and Hypothesis Development

### Self-Determination Theory

Self-determination theory, a psychological theory of motivation proposed by psychologists Deci and Ryan (2000), provides a new theoretical perspective for a more systematic explanation of human psychological needs, motivation and behavior and the relationship between the three. The term self-determination mainly refers to the individual’s psychological recognition of his or her own behavior in the process of activity (Knee et al., 2013). The theory believes that each person has the nature to find happiness, accept challenges and other positive growth, and the development of this nature depends on the support and promotion of the social environment, that is, a good social environment has a facilitating effect on the development of this nature, but a bad social environment can also hinder the development of nature (Deci et al., 2006; Knee et al., 2013). On the basis of this, Deci and Ryan (2000), after a lot of experimental research, concluded that psychological needs are necessary for each individual in the process of continuous growth and development, and that people have a variety of psychological needs, but the most basic needs are three, namely, autonomy, relatedness, and competence (Deci et al., 2006; Howard et al., 2020). Among them, AN refer to the individual’s ability to experience himself as the master of his own activities and the master of his own behavior; CN refer to the individual’s ability to experience himself as having a certain degree of competence in his activities; and relatedness needs refer to the individual’s ability to feel the care and support of others in the process of interaction with them. The mechanisms by which the social environment affects an individual’s growth and development are primarily through facilitating or hindering the satisfaction of these three basic needs of the individual (La Guardia et al., 2000; Howard et al., 2020).

People desire to establish and maintain relationships because of these basic psychological needs, and the social experience of need satisfaction contributes to the formation of attachment (Thomson, 2006). People desire to establish and maintain certain relationships because of these basic psychological needs, and the social experience of need satisfaction contributes to the formation of attachment (Thomson, 2006). In a marketing context, Thomson (2006) introduced self-determination theory to brand attachment research and found that the degree of satisfaction of autonomy and RN directly influenced the strength of consumer brand attachment, while the degree of satisfaction of competence needs did not have a significant effect on the strength of consumer brand attachment. According to this logic, this paper is devoted to a comprehensive analysis of the effect of these basic psychological needs on the emotional attachment between short video applications and users in the context of short video applications use.

### Hypothesis Development

#### Factors Influencing Emotional Attachment

Autonomy needs implies the pursuit of autonomous choice, autonomous control, and autonomous confirmation by individuals in their activities. Information systems, especially short video applications, in the context of the new wave of information technology can provide a variety of functions that allow participants to choose freely and encourage them to express themselves freely (La Guardia et al., 2000). The study found that the compromised autonomy of short video applications users prompted them to revise their judgment that a particular short video application does not satisfy the need for autonomy. In other words, there are many norms in the real world that prevent individuals from pursuing their pure “true selves.” Short video applications have always facilitated the realization of “true self” (Bargh et al., 2002), and over time, the accumulation of “true self” will be combined with a certain short video applications connection, i.e., the user’s cognitive reorganization and emotional attachment to short video applications. Clearly, the autonomy needs have a facilitating effect on emotional attachment (Gilal et al., 2017).

Relationship needs are the pursuit of interpersonal intimacy in people’s daily work life. Short video applications meet participants’ need for connection in two main ways: On the one hand, there is the satisfaction of general social bonding motivation. By establishing and maintaining connections with other people in short video applications, social–emotional support is obtained and loneliness is dispelled (McKenna and Bargh, 1999). Armstrong and Hagel (1996) have pointed out that short video applications can satisfy interpersonal needs and used virtual information systems as an example to show that the main purpose of users is to connect with each other and gain comfort. On the other hand, it is the establishment or adherence to new social norms within a certain context. The experience of intimacy and camaraderie is achieved by advocating for or participating in the establishment of certain reciprocal social norms within short video applications (Hars and Ou, 2002). In study of Fan (2017), forum moderators controlled the quality of posts and opposed “spamming,” and users practiced non-personalized gifting within short video applications with the aim of guiding the formation of group norms and experiencing more intimate interpersonal relationships. It is evident that relational demands have a facilitating effect on emotional attachment (Gilal et al., 2017).

Competence needs is the individual’s pursuit of efficiency, achievement, challenge, etc., in activities. Short video applications satisfy users’ competence needs in three main ways. The first aspect is the satisfaction of users’ instrumental motivations. For example, in an interview on short video applications users’ motivation for participation, “utility value motivation” was found to be the most frequently mentioned (Guo et al., 2019). In a subsequent empirical study, Chang and Yang (2009) verified the positive correlation between utility value and engagement behavior. The second aspect is the satisfaction of users’ achievement motivation. For example, chat rooms, forums, blogs, microblogs, etc., all provide space for real people who are not known in the real world to become “opinion leaders” in the network, thus creating an experience of effectiveness (Yang and Li, 2019). The third aspect is the satisfaction of users’ curiosity. For example, the games on Tik Tok provide almost all the features of a complex realistic society, in which users can try out multiple role plays and experience the richness and novelty of the characters (Li and Lu, 2007). The continuous satisfaction of users’ needs in short video applications leads to cognitive restructuring, which leads to the perception of short video applications as part of the self and emotional attachment (Gilal et al., 2017). It is evident that competence needs contribute to affective attachment. Based on the above reasoning, this study derives the following hypothesis:

*H1a*: Users’ autonomy needs is positively related to emotional attachment.

*H1b*: Users’ competence needs is positively related to emotional attachment.

*H1c*: Users’ relationship needs is positively related to emotional attachment.

#### Impact of Emotional Attachment on Relationship Quality

Relationship quality is a concept developed from relationship marketing. Relationship quality is a strategy and an important topic when it comes to relationship dynamics. In previous studies related to the literature, relationship quality has been an important indicator of the strength and goodness of the relationship between the customer and the company, and is an important outcome of relationship marketing. Crosby et al. (1990) pioneered the concept of relationship quality and defined it as how salespeople reduce uncertainty from the customer’s point of view, so that the customer trusts and has confidence in the salesperson’s future performance, which in turn affects the future the effectiveness of ongoing interactions. They argue that relationship quality is particularly important when customers are faced with many uncertainty risks resulting from factors such as intangibility, complexity, and lack of familiarity with the service. Sun et al. (2007) state that “these factors are very common for companies in the livestream e-commerce environment. Therefore, relationship quality is exceptionally important in livestream e-commerce scenarios.” In previous studies, relationship quality has been an important indicator of the strength and goodness of the user-firm relationship, and is an important outcome of relationship marketing (Smith, 1998). In contrast, affective attachment is a construct based on a relational process (Park et al., 2006). There is a sense in which there is an affective role from the relational process to a relational outcome state. Thus, emotional attachment as a relationship formation process may affect relationship quality as an outcome. The goal of short video applications is not only to provide users with virtual socialization, but its marketing focus is to build ongoing relationships with users through relationship marketing tools. Therefore, it is crucial for short video applications to enhance the quality of users’ relationships with short video applications through appropriate relationship tools (Wang and Head, 2007). Among the existing studies, few scholars have systematically explored the influence relationship between emotional attachment and relationship quality, but some studies have explored the effect of individual attachment on relationship quality related dimensions. Research of Thomson (2006) has shown that consumer attachment has a direct effect on relationship quality dimensions such as satisfaction, trust, and commitment. However, there is a lack of existing research examining the relationship between emotional attachment and relationship quality in the context of indigenous short video applications use. As a result, this paper proposes the following hypothesis.

*H2*: Users’ emotional attachment is positively related to relationship quality.

#### Mediating Role of Emotional Attachment

The relationship between emotional attachment in terms of psychological need and relationship quality is in line with the view of the theory of emotional expansion and construction. Fredrickson (1998) proposed the theory of emotional expansion and construction, which clarifies the mechanism of emotional action. The Broaden Mechanism, in which emotions transiently stimulate individual associations, promote creative thinking, and expand the range of action instruction systems and optional behaviors. Emotional attachment can stimulate positive experiential associations and put the user’s brain in a state of pleasure and inspiration for a short period of time, which in turn leads to creative use of short video applications and expands the frequency and quality of the user’s irrational engagement. Building mechanism, positive emotions promote personal resources through long-term learning, which in turn influences behavior. Emotional attachment promotes learning through different social cognitive channels, enhances personal resources, facilitates coping with problems encountered in using virtual communities, and ultimately increases the likelihood of long-term use.

First, in the context of short video applications use, autonomy needs are self-selected and self-controlled based on a balance of personal needs and environmental conditions, and are satisfied in the process. In the process of short video applications use, users desire to establish and maintain certain relationships because of autonomy needs, and the social experiences that need to be satisfied contribute to the formation of emotional attachments (Thomson, 2006). On the one hand, short video applications users are concerned about the negative experiences that are constantly eroding the original good memories, damaging the formed short video applications attachment, and causing the interruption or termination of the existing continuous use behavior. On the other hand, the entertainment and other content provided by short video applications enables users to satisfy practical values, while gaining sensory and psychological pleasure and enriching their selves, thus forming attachments to short video applications (Park et al., 2006). Users’ emotional attachment to short video applications will influence their attitudes toward short video applications (e.g., usage satisfaction, commitment, etc.), which in turn will influence usage propensity and behavior, thus contributing to the quality of the relationship between users and short video applications. Thus, emotional attachment plays a mediating role in the relationship of autonomy needs to relationship quality (Gilal et al., 2017).

Second, several times their own connection needs are met, short video applications users gradually discover that no matter how eccentric or niche their personality or needs are, they can find like-minded people through short video applications platforms. Short video applications can break the limits of time and space, linking participants to the same or similar individuals for solace. In addition, short video applications allow users to create new social order and behavioral norms in order to obtain the desired social emotions. In other words, users’ new cognitive judgments and memories of intimacy, belongingness, and support experiences formed by short video applications will gradually accumulate to cause qualitative changes, and users’ cognitive system will be reorganized, and the realization of self-relevant needs will be combined with a certain short video applications connection, i.e., users’ emotional attachment to short video applications will be formed. Emotional attachment will dominate the user’ s to maintain an ongoing relationship to short video applications after it is created. Therefore, affective attachment plays a mediating role in relationship needs on relationship quality (Gilal et al., 2017).

Third, in the process of satisfying their own competence needs, short video applications users gradually form new cognitive judgments, and short video applications is a new stage for self-expression, and the use of short video applications can realize competence needs. At the same time, short video applications users form and deepen memories of performance, achievement and novelty, and thus the cognitive system of short video applications users is reorganized, and the realization of self-competence needs is combined with short video applications, and short video applications becomes an integral part of the self. At this point, the quality of the relationship between the user and short video applications reaches its optimal state (Gilal et al., 2017). Thus, emotional attachment plays a mediating role in the relationship needs to relationship quality. In view of this, the following hypothesis is proposed in this study:

*H3a*: Users’ emotional attachment plays a mediating role between users’ autonomy needs and relationship quality.

*H3b*: Users’ emotional attachment plays a mediating role between users’ competence needs and relationship quality.

*H3c*: Users’ emotional attachment plays a mediating role between users’ relationship needs and relationship quality.

#### Moderating Role of Attachment Anxiety

Users are heterogeneous in their relationship building behavior with short video applications, i.e., not all users are eager or willing to maintain intimate and long-term relationships with short video applications (Thomson and Johnson, 2006). Therefore, short video applications that spends relationship-building investments on relationship-resistant users not only results in wasted resources, but also can even threaten the success of short video applications’ overall relationship marketing strategy. Identifying the right relationship-accepting users is undoubtedly of great value to the healthy development and sustainable operation of short video applications. Research on individual attachment trait types in attachment theory provides guidance for this purpose.

Brenan et al. (1998) found that there are two basic traits in adult attachment patterns: AA and attachment avoidance, and people with higher levels of attachment anxiety are more worried about whether their partner is approachable, responsive, and attentive, and this type of person is more willing to maintain an intimate relationship with their partner (Brenan et al., 1998). Thomson and Johnson (2006) found in a marketing scenario that anxiously attached individuals are more attentive and dependent on the reactions and behaviors of others and are more eager to establish an intimate relationship with business objects (Thomson and Johnson, 2006). This implies that high-anxiety individuals desire positive emotions in business relationships to balance out negative emotional experiences in purely interpersonal relationships.

From the above analysis, it is inferred that users with high levels of attachment anxiety have a greater impact on relationship quality when using short video applications with their level of emotional attachment. In contrast, users with low levels of relationship anxiety still have an effect of their level of emotional attachment on relationship quality, but not as significantly as users with high levels of relationship anxiety. Based on the above analysis, the following hypotheses are proposed in this paper:

*H4*: Users’ attachment anxiety plays a positively moderating role between users’ emotional attachment and relationship quality.

**Figure 1:**
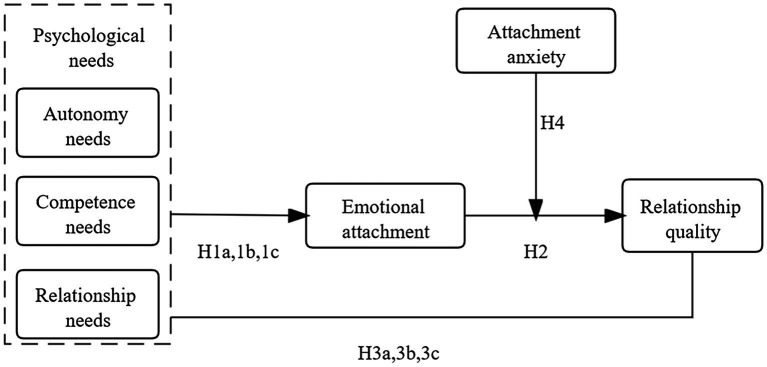
Theoretical model.

To evaluate our hypotheses, this study conducted an empirical study of short video applications users who had been using the service consistently for more than 6 months. Our theoretical model is depicted in Figure 1.

## Materials and Methods

### Samples

Participants were mainly university students recruited through links to questionnaires posted on WeChat and Weibo. This is because studies have shown that college students are the main users of short video applications, which is consistent with the representativeness of the sample survey. University students accessed the questionnaire through a hyperlink, and before answering the questionnaire, they were instructed to read an information sheet that told them participation was voluntary and anonymous, and that only users who had been using Tik Tok consistently for at least 6 months were eligible to university students. To ensure that participants were the target audience for this study, two filtering questions were used at the beginning of the survey to determine participant eligibility, i.e., exclusion of university students under the age of 18 and university students who had not been using consistently for 6 months. The survey period for this study was from September 18, 2021 to November 16, 2021. A total of 620 university students participated in the questionnaire, 8.06% of users (*n* = 50) did not answer the filtered questions correctly and were therefore excluded from the analysis. Around 512 valid samples were obtained after further processing of the questionnaires answered by 570 eligible university students and excluding invalid questionnaires.

### Measures

In order to ensure the reliability and validity of the measurement scales, the measurement questions in this study were all based on existing established scales, which were compiled through literature analysis according to the purpose of the study. Among them, in order to improve the applicability of the questionnaire, this study examined the matured scales according to the back-translation method. Moreover, to ensure the expert validity of the measurement questionnaire, this study invited three scholars in the field of short video applications research and three corporate executives to review the questionnaire, which was finally formed through the joint suggestions from the academic and practical communities. The variables were measured as follows.

Independent variables: Psychological needs. A 12-item scale, proposed by Chen (2015), were adopted to measure three dimensions of psychological needs, among which four items were used to measure autonomy needs, four items for measuring competence needs and four for assessing relationship needs. Emotional attachment. The emotional attachment scale is primarily based on the literature related to Thomson et al. (2005) and is modified into an emotional attachment measurement question based on the actual usage context of short video applications, which is mainly used to measure the emotional bond between users and short video applications, the scale has four question items. Attachment anxiety. The attachment anxiety scale in this study was based on Vlachos et al. (2010), and was modified to become a measure of attachment anxiety based on the actual context of short video applications use, mainly to measure the level of anxiety in maintaining long-term relationships between users and short video applications. It contains four questions. All scales are rated on a seven-point response scale ranging from 1 (strongly disagree) to 7 (strongly agree).

Dependent variables: Relationship quality. The relationship quality scale in this study mainly refers to research ideas of Smith (1998) and is modified into a relationship quality measurement question based on the actual usage context of short video applications, which is mainly used to measure the overall relationship strength formed by users’ perceptions toward short video applications, with five questions. This scale is rated on a seven-point response scale ranging from 1 (strongly disagree) to 7 (strongly agree).

Control variables. Previous studies and practices also show that gender, age, marital status, education, occupation, monthly consumption level, and time of use of short video applications users also influence their intention and behavior. For example, female users tend to use short video applications platforms for recreation and entertainment, while male users tend to use short video applications platforms to meet new friends or obtain useful information. Therefore, user characteristics such as gender, age, marital status, education, occupation, monthly consumption level, and time of use were included as control variables in this study, in order to provide a scientific and effective analysis of the internal logical relationships among other major constructs in this study.

### Data Analysis Method

In recent years, SEM has become the most popular method of data analysis in social sciences such as management, education, and psychology, and is considered a mainstream technique. SEM has become an important tool for testing theories with both experimental and non-experimental data (Jöreskog, 1971). From a statistical methodological perspective, SEM has the unique advantage of handling the data in a meticulous manner, allowing for both the prediction of the remaining estimated residuals and the separation of measurement errors, thus making SEM’s definition of latent variables more consistent with psychometric constructs. Therefore, this study used structural equation modeling software to test and analyze the hypothesized relationships and structural models in the study model. That is, the collected data were analyzed by SEM. Specifically, this study analyzed Confirmatory Factor Analysis (CFA), discriminant validity, structural model fit, and mediating and moderating effects using Mplus 7.0 programming software.

## Results

### Descriptive Statistical Analysis

The results of the descriptive statistical analysis of this study are shown in Table 1. Among them, the proportion of female users amounted to 57.62%, relatively more than men (42.38% were male). In terms of their age, users aged 18–23 (inclusive) are the most numerous, accounting for 35.55%; those aged 23–26 (inclusive) account for 32.03%, ranking second; those aged 27–30 (inclusive) account for 28.52%, ranking third; those aged 31 (inclusive) and above are the least numerous, accounting for 3.90%. In terms of their categories, 31.64% of students come from college, 33.20% from ordinary undergraduate schools, 19.92% from first-class universities and disciplines of the world, and 15.24% from key universities. In terms of its usage time, only 9.77% of users have been using it for more than 3 years, 32.42% of users have been using it for less than 1 year, and 57.81% of users have been using it for 1–2 years (inclusive).

**Table 1:** Descriptive statistical analysis.

Variables	Item	Frequency	%	Cumulative %
Gender	Male	217	42.38	42.38
	Female	295	57.62	100
Age	18–23 (inclusive)	182	35.55	35.55
	23–26 (inclusive)	164	32.03	67.58
	27–30 (inclusive)	146	28.52	96.10
	31 (inclusive) and above	20	3.90	100
Marriage	Married	20	3.91	3.91
	Unmarried	492	96.09	100
Education level	College	162	31.64	31.64
	Ordinary undergraduate schools	170	33.20	64.84
	First-class universities and disciplines of the world	102	19.92	84.76
	Key universities	78	15.24	100
Time	Less than 1 year	166	32.42	32.42
	1 ~ 2 years (inclusive)	296	57.81	90.23
	Over 3 years	50	9.77	100

### Confirmatory Factor Analysis

This study evaluates and revises the CFA measurement model based on a two-stage model (Kline, 2011). Currently, academics generally agree with the approach of Anderson and Gerbing (1998). That is, CFA should report Standardized Factor Loading (STD), Multivariate Correlation Squared, Composite Reliability (CR), and Average Variance Extracted (AVE) for all variables, and only after these metrics pass the test can structural models be evaluated. Specifically, STD is greater than 0.50, CR is greater than 0.60, and AVE is greater than 0.50 (Fornell and Lacker, 1981; Hair et al., 2017), then the measurement model has good convergent validity.

Table 2 reports the CFA of the measurement models. Among them, standardized factor loadings of all dimensions are between 0.619 and 0.941, and the composite reliability is between 0.822 and 0.915. Convergence Validity is between 0.539 and 0.731, indicating that each construct has good convergent validity.

**Table 2:** The analysis of confirmatory factor analysis (CFA).

Variables	Measurement items	Factor loading	CR	AVE
AN	AN1 Tik Tok makes me feel a sense of freedom	0.842	0.822	0.540
	AN2 Tik Tok is the social media I really want to use	0.778		
	AN3 Tik Tok allows me to be my most authentic self	0.619		
	AN4 I think Tik Tok is the social media I am really interested in	0.680		
CN	CN1 Tik Tok makes me feel confident	0.719	0.865	0.618
	CN2 Tik Tok makes me feel capable of doing what I want to do	0.750		
	CN3 Tik Tok makes me feel capable	0.787		
	CN4 Tik Tok makes me feel successful	0.878		
RN	RN1 I think Tik Tok cares about my experience	0.772	0.864	0.614
	RN2 I feel connected to Tik Tok	0.827		
	RN3 I think Tik Tok is important to me	0.771		
	RN4 I think Tik Tok makes me feel warm	0.763		
RQ	RQ1 I had a delightful experience on Tik Tok	0.649	0.853	0.539
	RQ2 Tik Tok meets my needs well	0.790		
	RQ3 Tik Tok provides a good service	0.852		
	RQ4 The information provided by Tik Tok is trustworthy	0.650		
	RQ5 Overall, I am satisfied with Tik Tok	0.709		
EA	EA1 My feelings for Tik Tok are genuine	0.672	0.836	0.565
	EA2 I am emotionally connected to Tik Tok	0.818		
	EA3 I am passionate about using Tik Tok	0.857		
	EA4 I am emotionally attached to Tik Tok	0.635		
AA	AA1 I often worry that people close to me do not understand my needs	0.701	0.915	0.731
	AA2 I have a lot of concerns about my various relationships	0.905		
	AA3 As a user, I worry about being abandoned by Tik Tok	0.941		
	AA4 As a user, I am worried that Tik Tok is not really looking out for me	0.853		

AN, autonomy needs; CN, competence needs; RN, relationship needs; EA, emotional attachment; RQ, relationship quality; and AA, attachment anxiety.

### Discriminant Validity

Discriminant validity is a measure to test whether any two variables in a theoretical model are identical to each other. The interrelationship between the two constructs in this study and the discriminant validity are shown in Table 3. This study uses the latest discriminant validity analysis method, namely CI method (Torkzadeh et al., 2003). The CI method is used to confirm the CI of the correlation coefficient between variables. If it fails to include “1,” then it is completely correlated, indicating that the facets have different validity. As suggested, bootstrap test was conducted in this study. About 95% CI of the correlation coefficient does not involve 1 (see Table 3), which shows the good discriminant validity between all the variables. Therefore, the measurement model has good discriminative validity.

**Table 3:** The analysis of discriminant validity.

Parameter	Correlation coefficients	Bias-corrected 95%	
		Lower	Upper
AN<-->CN	0.534	0.440	0.637
AN<-->RN	0.352	0.229	0.468
AN<-->EA	0.511	0.362	0.652
AN<-->AA	0.159	0.048	0.275
AN<-->RQ	0.478	0.361	0.584
CN<-->RN	0.493	0.343	0.607
CN<-->EA	0.520	0.396	0.626
CN<-->AA	0.097	−0.020	0.225
CN<-->RQ	0.518	0.385	0.630
RN<-->EA	0.491	0.393	0.579
RN<-->AA	0.113	0.001	0.213
RN<-->RQ	0.411	0.298	0.507
EA<-->AA	0.087	−0.015	0.196
EA<-->RQ	0.621	0.435	0.761
RQ<-->AA	0.010	−0.100	0.119

AN, autonomy needs; CN, competence needs; RN, relationship needs; EA, emotional attachment; RQ, relationship quality; and AA, attachment anxiety.

### Model Fit Degree

The study by Jackson et al. (2009) concluded that in structural models, model fit metrics should be reported as a way to assess, correct, and judge the goodness of measurement models. According to Jackson et al. (2009) criteria, nine goodness-of-fit metrics are usually used to test the model fit. In principle, the lower the *χ*^2^, the better, but since *χ*^2^ is very sensitive to sample size, the ideal value of *χ*^2^/df should be less than 3. The criteria for all indicators are shown in Table 4. The results of this study based on Jackson et al. (2009) criteria and applying Amos 24.0 for the analysis are shown in Table 4, all of which met the criteria. Therefore, the structural model of this study has a good model fit.

**Table 4:** Model fit criteria and the test results.

Model fit	Criteria	Model fit of research model	Result
*χ* ^2^	The small the better	225.282	
DF	The large the better	182	
Normed Chi-square(*χ*^2^/DF)	1 < *χ*^2^/DF < 3	1.238	Pass
RMSEA	<0.08	0.022	Pass
SRMR	<0.08	0.069	Pass
TLI (NNFI)	>0.9	0.991	Pass
CFI	>0.9	0.992	Pass
GFI	>0.9	0.962	Pass
AGFI	>0.9	0.948	Pass

### Regression Coefficient

The regression coefficients are reported in Table 5. In this study, the path coefficient relationships of the measurement models are shown in Table 5. Specifically, AN (*β* = 0.253, *p* < 0.001), CN (*β* = 0.235, *p* < 0.001), and RN (*β* = 0.248, *p* < 0.001) positively affect the EA. EA (*β* = 0.610, *p* < 0.001) positively affect RQ. Therefore, H1a, H1b, H1c, and H2 are supported.

**Table 5:** The analysis of regression coefficient.

Path	Unstd	S.E.	Unstd./S.E.	*t*-value
*H1*a: AN->EA	0.253	0.046	5.456	0.303
*H1*b: CN->EA	0.235	0.054	4.308	0.253
*H1*c: RN->EA	0.248	0.047	5.317	0.278
*H2*: EA->RQ	0.610	0.057	10.644	0.658

AN, autonomy needs; CN, competence needs; RN, relationship needs; EA, emotional attachment; and RQ, relationship quality.

### Mediating Effect Analysis

In this study, structural equation modeling was used to analyze the mediating effect, and the SE of the mediating effect was first estimated using Bootstrap estimation technique, and then the significant level of the mediating effect was further calculated. According to Hayes (2009), a mediating effect is indicated if “0” does not include the 95% CI of Bias-corrected, the z-value is greater than 1.96, and the value of *p* is less than 0.05.

Specifically, the mediating effects were analyzed as shown in Table 6. The total effect of autonomy needs on relationship quality is 0.202. At the 95% confidence level, “0” does not include the Bias-corrected 95% CI range, the z-value is greater than 1.96, and the value of *p* is less than 0.05. Therefore, there is a total effect exists. The indirect effect is 0.095, “0” does not include the Bias-corrected 95% CI range, the z-value is greater than 1.96, and the value of *p* is less than 0.05. Therefore, there is an indirect effect. The direct effect is 0.107, “0” include the Bias-corrected 95% CI range, the z-value is not greater than 1.96, and the value of *p* is not less than 0.05. Therefore, there is not a direct effect exists. Therefore, H3a is established and is a partial mediation.

**Table 6:** The analysis of mediation effect.

Effect	Point estimate	Product of coefficients			Bootstrap	
					Bias-corrected 95%	
		SE	z-value	*p*-value	Lower	Upper
Total effect: AN→RQ	0.202	0.061	3.311	0.002	0.088	0.330
Indirect effect: AN→EA→RQ	0.095	0.045	2.111	0.003	0.028	0.198
Direct effect: AN→RQ	0.107	0.075	1. 427	0.106	−0.023	0.330
Total effect: CN→RQ	0.251	0.077	3.260	0.005	0.076	0.386
Indirect effect: CN→EA→RQ	0.081	0.039	2.077	0.004	0.025	0.180
Direct effect: CN→RQ	0.169	0.076	2.224	0.045	0.003	0.307
Total effect: RN→RQ	0.144	0.057	2.893	0.005	0.040	0.268
Indirect effect: RN→EA→RQ	0.095	0.039	2.875	0.005	0.028	0.180
Direct effect: RN→RQ	0.049	0.064	3.186	0.366	−0.061	0.200

AN, autonomy needs; CN, competence needs; RN, relationship needs; EA, emotional attachment; and RQ, relationship quality.

The total effect of competence autonomy needs on relationship quality is 0.251. At the 95% confidence level, “0” does not include the bias-corrected 95% CI range, the z-value is greater than 1.96, and the value of *p* is less than 0.05. Therefore, there is a total effect exists. The indirect effect is 0.081, “0” does not include the Bias-corrected 95% CI range, the z-value is greater than 1.96, and the value of *p* is less than 0.05. Therefore, there is an indirect effect. The direct effect is 0.169, “0” does not include the bias-corrected 95% CI range, the z-value is greater than 1.96, and the value of *p* is less than 0.05. Therefore, there is not an direct effect exists. Therefore, H3b is established and is a partial mediation.

The total effect of relationship needs on relationship quality is 0.144. At the 95% confidence level, “0” does not include the Bias-corrected 95% CI range, the z-value is greater than 1.96, and the value of *p* is less than 0.05. Therefore, there is a total effect exists. The indirect effect is 0.095, “0” does not include the bias-corrected 95% CI range, the z-value is greater than 1.96, and the value of *p* is less than 0.05. Therefore, there is an indirect effect. The direct effect is 0.049, “0” include the bias-corrected 95% CI range, the z-value is not greater than 1.96, and the value of *p* is not less than 0.05. Therefore, there is not an direct effect exists. Therefore, H3c is established and is a fully mediation. Therefore, H3a, H3b, and H3c are supported.

### Moderating Effect Analysis

**Figure 2:**
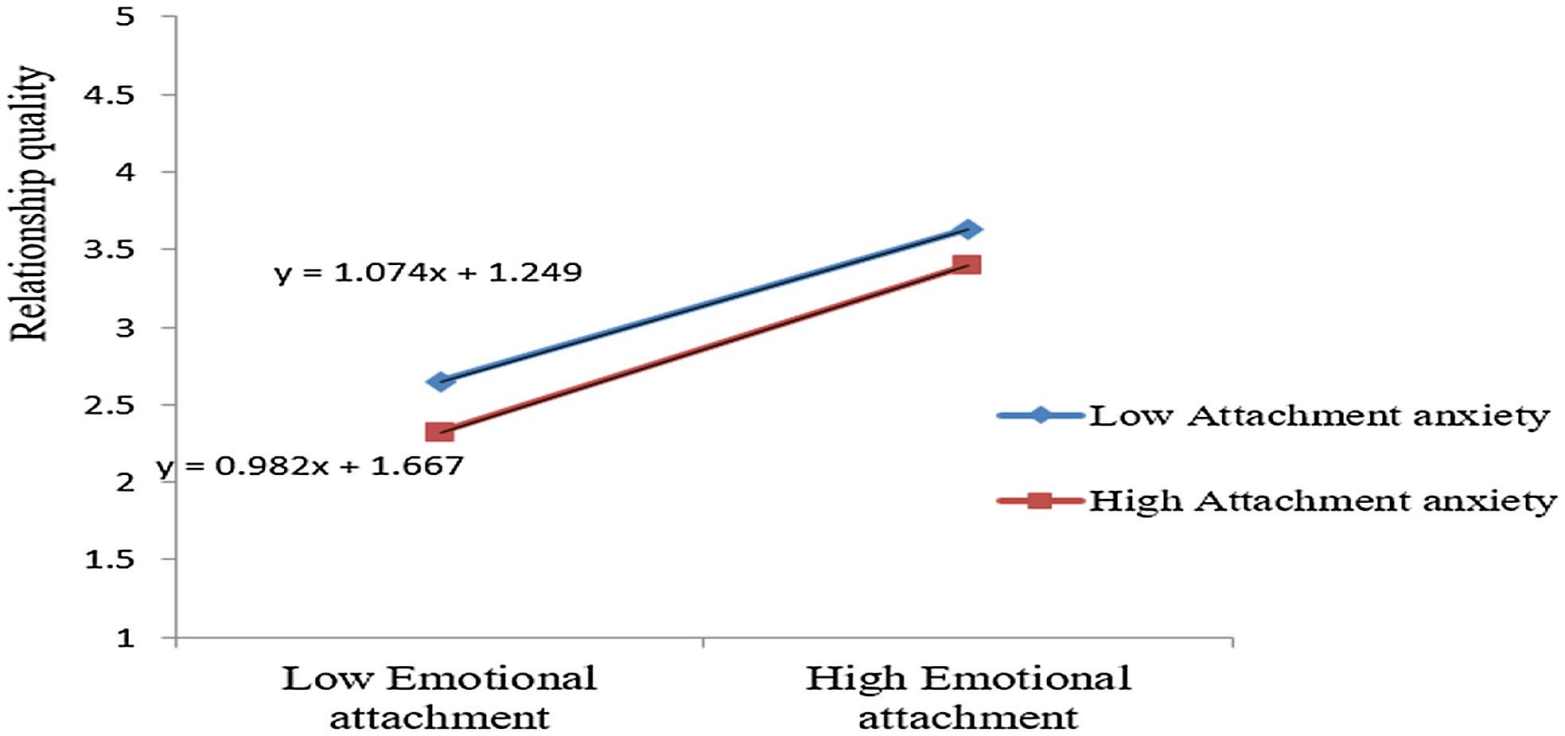
*Attachment anxiety* (AA) moderating the relationship between emotional attachment (EA) and relationship quality (RQ).

The moderating effects are reported in Table 7. In the present study, *AA* is the moderating variable. The results of structural equation modeling have been shown that the moderator effect of emotional attachment × *attachment anxiety* on relationship quality is 0.023 (z = |2.468| > 1.96, *p* = 0.014 < 0.05), implying the presence of a positive moderating effect of *AA* on the relationship between EA and RQ. Specifically, the slope of EA on RQ increases positively by 0.067 units for each 1-unit increase in the moderating variable AA. That is, AA has a positive moderating effect. Therefore, hypothesis 5 is verified. The result is shown in Figure 2.

**Table 7:** The analysis of moderating effect.

Dependent variable (DV)	Independent variable (IV)	Path coefficient (**β**)	S.E.	z-value	*p*
RQ	EA	0.514	0.058	8.816	0.000
	AA	−0.140	0.053	−2.631	0.009
	EA × AA	0.023	0.009	2.468	0.014

RQ, relationship quality; EA, emotional attachment; and AA, attachment anxiety.

## Research Results and Discussion

### Research Results

First, the role of psychological needs in influencing emotional attachment was confirmed. The results of the empirical analysis showed that all three antecedent variables of affective attachment, autonomy needs, competence needs, and relationship needs, had a significant positive effect on affective attachment. In previous attachment theory studies, the antecedents of attachment have rarely been examined systematically, and there is a lack of holistic studies that simultaneously examine the antecedent outcomes of emotional attachment in a single study. The study in this paper fills this theoretical gap. This paper identifies and examines the factors influencing emotional attachment, including autonomy needs, competence needs, and relationship needs. It is hypothesized that the reason for this is that the current short video applications are booming and widely used, and its social context is obviously more different from the virtual environment in the broad sense, except for the similarities, such as the user characteristics of liking, commenting and retweeting, and the boundary between “virtual-reality” is constantly conflicting in the short video applications usage context, and the relationship between users and short video applications is closer.

Second, the significant role of consumer emotional attachment on relationship quality formation was verified. Relationship quality is an important indicator of the strength and goodness of the relationship between users and short video applications. The results of the empirical test in this paper found that users’ emotional attachment has a significant positive effect on the quality of their relationship with short video applications. This suggests that emotional attachment is an important factor in enhancing the quality of the relationship between users and short video applications and investigates the persistence mechanism of the relationship between users and short video applications from a relational perspective, which provides a useful supplement to the lack of explanatory power of existing related studies, which are mostly conducted from a transactional perspective. This theoretically validates thesis of that “attachment predicts higher-level loyalty behaviors such as customer commitment,” highlighting the importance of attachment theory in the formation of relationship quality in the context of short video applications use, and also answering the question of how emotional attachment affects relationship quality in the context of short video applications use. It also answers the question of the mechanism of the influence of emotional attachment on relationship quality in the context of short video applications use.

Third, it was confirmed that the level of attachment anxiety was a moderating variable between emotional attachment and relationship quality. The results of the study show that the level of user attachment anxiety has a significant positive moderating effect on the relationship between emotional attachment and relationship quality, thus revealing the boundary conditions of the relationship between emotional attachment and relationship quality. Especially in the context of artificial intelligence, cloud computing, blockchain, and the continuous intensification of competition, studying the influence of emotional attachment on users’ persistent use behavior, the antecedents and consequences of virtual information system attachment mechanism of action, etc., can help to understand the formation mechanism of individual persistent use behavior more completely. In recent years, attachment theory has been a hot topic of attention in both theoretical and practical circles and has become a research hotspot for further theoretical enrichment and development in the fields of brand attachment, local attachment, and adult attachment. This study, however, applies attachment anxiety as a moderating variable in the local short video applications use context to explore the relationship quality of the continuous use behavior between users and short video applications, thus verifying the differences in the relationship quality between consumers and short video applications.

### Theoretical Contributions

Firstly, a theoretical model of psychological needs predicting user relationship quality was constructed. Based on the Chinese contextual perspective, this study constructs a theoretical model of psychological needs predicting user relationship quality, discusses in depth the relationship between different components of psychological needs, emotional attachment, and user relationship quality, and discovers the different psychological needs patterns generated by users’ continuous use of short video applications, namely, autonomy needs, competence needs and this study found out the role and mechanism of autonomy needs, competence needs and relationship needs in driving relationship quality. The results of structural equation modeling analysis showed that different components of psychological needs can influence users’ relationship quality through the indirect effect of emotional attachment, and emotional attachment has a mediating role between psychological needs and relationship technician, which also further validates the explanatory validity of psychological needs on relationship quality.

Secondly, in this paper, emotional attachment and relationship quality are discussed together in a cross-disciplinary study. Research on relationship quality is currently focused on the field of marketing, while emotional attachment is mainly applied to psychology-related research. Although the influence of psychological needs, the antecedent variable of affective attachment, is more evident from a practical perspective, and although research on the relationship between relationship quality and affective attachment has attracted attention based on analysis of the theoretical literature, there is still a lack of sufficient evidence. Therefore, this study breaks through the shortcomings of previous studies, which are mainly limited to within their respective fields, and focuses explicitly on the relationship between emotional attachment and relationship quality, verifying the influence of emotional attachment on relationship quality through empirical studies, and exploring the underlying deep influence mechanisms from a cross-disciplinary perspective.

Thirdly, this study applied attachment anxiety as a moderating variable to short video applications use contexts, confirming the variability that exists in individuals’ relationships with short video applications to establish attachments. By introducing privacy-focused attachment anxiety as a moderating variable into short video applications use contexts, this study extends existing research on the quality of relationships and intermediate mechanisms by which emotional attachment affects relationships, and confirms the existence of differences in the relationships between users and short video applications to establish ongoing use mechanisms. The results of the empirical study showed that individuals’ attachment anxiety levels had a significant positive moderating effect on the relationship between emotional attachment and relationship quality. This suggests that as individuals’ attachment anxiety levels increase, the effect of emotional attachment on relationship quality also increases. This study enriches the boundary conditions for the effect of emotional attachment on relationship quality.

### Practical Implications

First, emotional attachment is a decisive condition for building a relationship for sustained use by short video applications users. This study shows that in the context of short video applications use, emotional attachment is a direct stimulus and mediating variable for users’ continued use of the relationship, so short video applications should elevate caring for users and building emotional connections with them to a strategic level. The results of this paper suggest that short video applications that are willing to invest in building an emotional connection between individuals and short video applications are likely to reap the benefits of continued user use and the resulting high-quality quality relationships. Therefore, short video applications can provide hedonic, functional, and symbolic resources to satisfy, realize, and enrich the self, and provide products and services to users with a meticulous service attitude and behavior to continuously stimulate the emotional bond between users and short video applications, and stimulate users to pay personal resources to short video applications, thus causing users to undergo cognitive restructuring, linking users’ self with short video applications, developing emotional attachment, and generating users’ continuous usage behavior characterized by investing personal resources to the short video applications they are attached to. Invest enough in the emotional ties between users and short video applications to continuously improve the ability to acquire new users and retain old ones, and maintain a continuous relationship between users and short video applications use. Adopt differentiated marketing strategies, provide users with products and services that meet their psychological needs, optimize product functionality and interface design, care for users’ emotional needs, win them to develop an emotional preference for a particular short video application, and play the role of direct driver of emotional attachment to user relationships.

Second, psychological needs are an important driver to stimulate users’ emotional attachment. Short video applications should be adept at using the key elements of building emotional connections with users. The important antecedents of emotional attachment identified in this paper will guide individuals in where to invest to build emotional connections with individuals and thus help short video applications to develop effective user retention strategies. Specifically, short video applications should build this connection by investing in the immediate antecedents that influence emotional attachment. In addition to focusing on improving the functionality and speed of product search and increasing the autonomy needs of short video applications, which are the most basic factors of importance, short video applications should focus on investing in enhancing the technical support of user-oriented online services, increasing the entertainment value of short video applications, and creating the need for association in order to build emotional connections with individuals. Due to the important role of user-orientation in forming emotional attachments to individuals, short video applications should meet the needs of quality customers based on a high degree of customization and personalization, which requires the following: ensuring a high level of user support and services, especially for the “most valuable” and “most promising” users; providing online customer service and technical support 24 h a day, 7 days a week; achieving customer differentiation and creating barriers to entry for competitors, etc.

Third, segmentation of target markets is an effective way to classify and manage the different needs of different groups. This study shows that attachment anxiety can influence the positive impact of emotional attachment on relationship quality, suggesting that short video applications should implement market segmentation and develop targeted relationship marketing strategies based on user traits. This paper shows that high anxiety users’ emotional attachment has a stronger impact on the quality of their relationships with short video applications. Thus, short video applications should focus their investment mainly on this group and try to meet the emotional attachment needs of users with high attachment anxiety levels. For the remaining individuals with low levels of attachment anxiety, the effect of emotional attachment on relationship quality may be more modest. Thus, the same effect may not be achieved for this group of users through rich emotional mechanisms. Based on this, short video applications may consider investing in factors that are included in the traditional satisfaction-loyalty relationship marketing paradigm (e.g., ease of use, usefulness, etc.). In addition, the discovery of the role of attachment anxiety has important implications for short video applications in organically coordinating the user acquisition and user retention phases and rationalizing resource investment. In the user acquisition stage, securely attached users are more likely to trust short video applications and are more satisfied with the relationship than non-securely attached users such as anxious and avoidant users. Therefore, it is suggested that the investment of short video applications in the user acquisition stage is from high to low: avoidant, anxious, and secure. In the user retention stage, because anxiously attached individuals are highly dependent on other people’s reactions and behaviors, they are more willing to immerse themselves in the relationship after establishing a close relationship with short video applications, stick to short video applications, and show higher satisfaction, trust and commitment to short video applications. Compared with other types of users, short video applications can retain high anxiety attachment users at a relatively low cost. Therefore, it is suggested that short video applications should invest in the retention phase from high to low: avoidant, secure, and anxious. The above analysis provides useful references for companies to adopt corresponding relationship management strategies for different traits of consumer groups.

### Research Limitations and Future Research Directions

This study suffers from data statics and cross-sectional limitations. Although the collected data provide initial support for the hypothesis, its key variables are self-reported at the same time and thus the results are subject to common method bias, which may increase the reported effect size despite statistically validated small effects (Podsakoff et al., 2012). Furthermore, although hypothesis testing has well-established a positive relationship between psychological needs and relationship quality, it is still not possible to infer the direction of the relationship between psychological needs and relationship quality from cross-sectional data. Therefore, in future studies, these limitations can be addressed using a two-wave online panel sample and a dynamic tracking method to examine the trend of users’ psychological needs and relationship quality in order to enhance the persuasiveness and rigor of the study findings.

In addition, in terms of research variables. The influence of psychological needs on relationship quality is somewhat conditional, and this study mainly. This study mainly investigated the mechanism of the moderating effect of attachment anxiety on the relationship between emotional attachment and relationship quality, while the potential factors that may affect the effect of relationship quality, such as the user’s personality characteristics and the type of short video application, were not all examined. The present study mainly investigated the moderating mechanism of attachment anxiety on the relationship quality, but not all the potential factors that may affect the effect of relationship quality, such as user personality characteristics and short video application type, were examined. Therefore, the future studies can further investigate the moderating effects of potential variables. For example, the moderating effects of consumption level, education level, and economic development level can be investigated. The moderating effects of consumption level, education level, and economic development level can be examined, and these potential moderating variables can be analyzed to further validate and enrich the theoretical model proposed in this study. The theoretical model proposed in this study can be further validated and enriched by analyzing these potential moderating variables.

## Data Availability Statement

The raw data supporting the conclusions of this article will be made available by the authors, without undue reservation.

## Ethics Statement

Ethical review and approval were not required for the study on human participants in accordance with the local legislation and institutional requirements. Informed consent was obtained from all subjects involved in the study.

## Author Contributions

ZH, LZ, and JZ contributed to conceptualization and methodology, formal analysis, investigation, and visualization. ZH, LZ, JZ, SK, and WC contributed to writing about original draft preparation and review and editing. All authors contributed to the article and approved the submitted version.

## Funding

This study appreciated the partial financial support from Soft Science Project of Science and Technology Department in Henan Province in 2022 (Study on the active inheritance of intangible cultural heritage of traditional Villages in the Yellow River Basin of Henan Province in the context of rural revitalization, Grant No. 222400410385).

## Conflict of Interest

The authors declare that the research was conducted in the absence of any commercial or financial relationships that could be construed as a potential conflict of interest.

## Publisher’s Note

All claims expressed in this article are solely those of the authors and do not necessarily represent those of their affiliated organizations, or those of the publisher, the editors and the reviewers. Any product that may be evaluated in this article, or claim that may be made by its manufacturer, is not guaranteed or endorsed by the publisher.

## References

[ref1] Ahmed R. R. Hussain S. Pahi M. H. Usas A. Jasinskas E. (2019). Social media handling and extended technology acceptance model (ETAM): evidence from SEM-based multivariate approach. Transform. Bus. Econ. 18, 246–271.

[ref2] Al-Debei M. M. Al-Lozi E. Papazafeiropoulou A. (2013). Why people keep coming back to Facebook: explaining and predicting continuance participation from an extended theory of planned behaviour perspective. Decis. Support. Syst. 55, 43–54. doi: 10.1016/j.dss.2012.12.032

[ref300] Anderson J. C. Gerbing D. W. (1998). Structural equation modeling in practice, A review and recommended two-step approach. Psychological Bulletin. 10, 411–423. doi: 10.1037/0033-2909.103.3.411

[ref3] Armstrong A. Hagel J. (1996). The real value of on-line communities. Harv. Bus. Rev. 74, 134–141. doi: 10.1016/B978-0-7506-9850-4.50007-5

[ref5] Bargh J. A. McKenna K. Y. A. Fitzsimons G. M. (2002). Can you see the real me? Activation and expression of the "true self" on the internet. J. Soc. Issues 58, 33–48. doi: 10.1111/1540-4560.00247

[ref7] Brenan K. A. Clark C. L. Shaver P. R. (1998). “Selfreport measurement of adult attachment: an integrative overview,” in Attachment Theory and Close Relationships. eds. Sinson J. A. Rholes W. S. (New York: Guilford Press), 46–76.

[ref8] Camacho D. Luzon M. V. Cambria E. (2021). New research methods & algorithms in social network analysis. Futur. Gener. Comput. Syst. 114, 290–293. doi: 10.1016/j.future.2020.08.006, PMID: 35219277

[ref9] Casalo L. V. Romero J. (2019). Social media promotions and travelers' value-creating behaviors: the role of perceived support. Int. J. Contemp. Hosp. Manag. 31, 633–650. doi: 10.1108/IJCHM-09-2017-0555

[ref400] Chang J. Yang J. M. (2009). An empirical study on the relationship between participation behavior and participation motivation of Baidu Encyclopedia users. Scientific Research. 27, 1213–1219. doi: 10.16192/j.cnki.1003-2053.2009.08.021

[ref200] Chen X. M. (2015). Application and exploration of grounded theory in education research. Peking University Educ. Rev. 2–15. doi: 10.19355/j.cnki.1671-9468.2015.01.002, PMID: 21850154

[ref10] Chen J. Kou G. Peng Y. Chao X. R. Xiao F. Alsaadi F. E. (2020). Effect of marketing messages and consumer engagement on economic performance: evidence from Weibo. Internet Res. 30, 1565–1581. doi: 10.1108/INTR-07-2019-0296

[ref11] Crosby L. A. Evans K. R. Cowels D. (1990). Relationship quality in service selling: an interpersonal influence perspective. J. Mark. 54, 68–81. doi: 10.1177/002224299005400306

[ref12] Deci E. L. La Guardia J. G. Moller A. C. Scheiner M. J. Ryan R. M. (2006). On the benefits of giving as well as receiving autonomy support: mutuality in close friendships. Personal. Soc. Psychol. Bull. 32, 313–327. doi: 10.1177/0146167205282148, PMID: 16455859

[ref13] Deci E. L. Ryan R. M. (2000). The "what" and "why" of goal pursuits: human needs and the self-determination of behavior. Psychol. Inq. 11, 227–268. doi: 10.1207/S15327965PLI1104_01, PMID: 20204932

[ref14] Fan J. (2017). The impact of WeChat public accounts' push content characteristics on users' continuance intention. J. Bus. Econ. 8, 69–78. doi: 10.14134/j.cnki.cn33-1336/f.2017.08.007

[ref15] Fornell C. R. Lacker D. F. (1981). Structural equation models with unobservable variables and measurement error. J. Mark. Res. 18, 382–388. doi: 10.1177/002224378101800313

[ref16] Fredrickson B. L. (1998). What good are positive emotions. Rev. Gen. Psychol. 2, 300–319. doi: 10.1037/1089-2680.2.3.300, PMID: 21850154 PMC3156001

[ref17] Gan C. M. Huang K. Xu J. Y. Lin T. T. (2018a). The influences of technological factors and perceived values on continuance intention of social commerce: an empirical study. Data Anal. Knowl. Discov. 2, 29–37. doi: 10.11925/infotech.2096-3467.2017.1250

[ref18] Gan C. M. Liang X. B. Li T. (2018b). A review of social network user behavior research from the perspective of use and satisfaction: a content analysis based on 54 foreign empirical research papers. Library Intell. Work 62, 134–143. doi: 10.13266/j.issn.0252-3116.2018.07.016

[ref19] Gilal F. G. Jian Z. Gilal R. G. Gilal N. G. (2017). Supply chain management practices and product development: a moderated mediation model of supply chain responsiveness, organization structure, and research and development. J. Adv. Manuf. Syst. 16, 35–56. doi: 10.1142/S0219686717500032

[ref20] Gong Y. P. Cao Y. Li J. (2020). The influence of characteristics of short video applications on users' engagement behavior: the mediating role of psychological engagement. Intell. Sci. 38, 77–84. doi: 10.13833/j.issn.1007-7634.2020.07.011

[ref21] Guede J. R. S. Filipe A. J. F. (2019). The electronic brand experience through social media and its influence on the electronic relationship quality and electronic loyalty. Empirical analysis on travel websites. Cuadernos De Turismo 44, 351–380. doi: 10.6018/turismo.44.404891

[ref22] Guo Y. G. Tianyong W. Wu H. Chou S. (2019). Research on social network opinion leaders: butterfly schema, identification and impact assessment. Library Inform. Serv. 63, 62–73. doi: 10.13266/j.issn.0252-3116.2019.14.008

[ref23] Hair J. F. Hult G. T. M. Ringle C. M. Sarstedt M. Thiele K. O. (2017). Mirror, mirror on the wall: a comparative evaluation of composite-based structural equation modeling methods. J. Acad. Mark. Sci. 45, 616–632. doi: 10.1007/s11747-017-0517-x

[ref24] Hars A. Ou S. (2002). Working for free? Motivations for participating in open-source projects. Int. J. Electron. Commer. 6, 23–37.

[ref25] Hayes A. F. (2009). Beyond baron and kenny: statistical mediation analysis in the new millennium. Commun. Monogr. 76, 408–420. doi: 10.1080/03637750903310360

[ref26] Hayes C. Stott K. Lamb K. J. Hurst G. A. (2020). "Making every second count": utilizing TikTok and systems thinking to facilitate scientific public engagement and contextualization of chemistry at home. J. Chem. Educ. 97, 3858–3866. doi: 10.1021/acs.jchemed.0c00511

[ref27] Howard J. L. Gagne M. Morin A. J. S. (2020). Putting the pieces together: reviewing the structural conceptualization of motivation within SDT. Motiv. Emot. 44, 846–861. doi: 10.1007/s11031-020-09838-2

[ref28] Hsu M. H. Chang C. M. Chuang L. W. (2015). Understanding the deter minants of online repeat purchase intention and moderating role of habit: the case of online group-buying in Taiwan. Int. J. Inf. Manag. 35, 45–56. doi: 10.1016/j.ijinfomgt.2014.09.002

[ref29] Hu J. Zhang Y. (2016). Understanding Chinese undergraduates' continuance intention to use mobile book reading apps: an integrated model and empirical study. Libri 66, 85–99. doi: 10.1515/libri-2015-0090

[ref30] Jackson D. L. Gillaspy J. A. Purc-Stephenson R. (2009). Reporting practices in confirmatory factor analysis: an overview and some recommendations. Psychol. Methods 14, 6–23. doi: 10.1037/a0014694, PMID: 19271845

[ref31] Jöreskog K. G. (1971). Statistical analysis ofsets of congeneric tests. Psychometrika 36, 109–133. doi: 10.1007/BF02291393

[ref32] Kline R. B. (2011). Principles and Practice of Structural Equation Modeling. 3rd Edn. New York: Guilford.

[ref33] Knee C. R. Hadden B. W. Porter B. Rodriguez L. M. (2013). Self-determination theory and romantic relationship processes. Personal. Soc. Psychol. Rev. 17, 307–324. doi: 10.1177/1088868313498000, PMID: 23921674

[ref34] La Guardia J. G. Ryan R. M. Couchnan C. E. (2000). Within-person variation in security of attachment: a self determination theory perspective on attachment, need-fulfillment and well-being. J. Pers. Soc. Psychol. 79, 367–384. doi: 10.1037/0022-3514.79.3.367, PMID: 10981840

[ref500] Li Y. F. Lu X. W. (2007). An Empirical Study of Online Games on the Motivation of Virtual Community Members. Nankai business Review. 5, 55–60.

[ref35] Li Z. X. Zhang S. M. Liu H. B. Wu Q. T. (2021). User characteristics & service characteristics’ influence on user satisfaction and continuous use intention in short video platform: an example of Chinese TikTok users during COVID pandemic period. J. China Stud. 24, 45–64. doi: 10.20288/JCS.2021.24.2.45, PMID: 35215814

[ref36] Liquan Yoon S. (2021). An empirical study on the determinant factors affecting customer's brand citizenship behaviour in social media. J. Prod. Res. 37, 123–133. doi: 10.36345/kacst.2019.37.1.014

[ref37] Ma X. M. Sun Y. Q. Guo X. T. Lai K. H. Vogel D. (2021). Understanding users' negative responses to recommendation algorithms in short-video platforms: a perspective based on the stressor-strain-outcome (SSO) framework. Electron. Mark. 1–24. doi: 10.1007/s12525-021-00488-x

[ref38] McKenna K. Y. A. Bargh J. A. (1999). Causes and consequences of social interaction on the internet: a conceptual framework. Media Psychol. 1, 249–269. doi: 10.1207/s1532785xmep0103_4, PMID: 34916999

[ref39] Meng K. S. Leung L. (2021). Factors influencing TikTok engagement behaviors in China: an examination of gratifications sought, narcissism, and the big five personality traits. Telecommun. Policy 45:102172. doi: 10.1016/j.telpol.2021.102172

[ref40] Mou X. B. Xu F. Du J. T. (2021). Examining the factors influencing college students' continuance intention to use short-form video APP. Aslib J. Inf. Manag. 73, 992–1013. doi: 10.1108/AJIM-03-2021-0080

[ref41] Oghuma A. P. Libaque-Saenz C. F. Wong S. F. Chang Y. (2016). An expectation-confirmation model of continuance intention to use mobile instant messaging. Telematics Inform. 33, 34–47. doi: 10.1016/j.tele.2015.05.006

[ref42] Park C. W. Macinnis D. J. Priester J. (2006). Beyond attitudes: attachment and consumer behavior. Seoul J. Bus. 12, 3–35. doi: 10.1016/j.jcps.2008.09.006

[ref43] Podsakoff P. M. MacKenzie S. B. Podsakoff N. P. (2012). Sources of method bias in social science research and recommendations on how to control it. Annu. Rev. Psychol. 63, 539–569. doi: 10.1146/annurev-psych-120710-100452, PMID: 21838546

[ref44] Smith J. B. (1998). Buyer-seller relationships: similarity, relationship management, and quality. Psychol. Mark. 15, 3–21. doi: 10.1002/(SICI)1520-6793(199801)15:1<3::AID-MAR2>3.0.CO;2-I

[ref45] Sun P. Shao S. Shi J. Y. Kang W. Q. (2020). Formation mechanism of Tik Tok users' travel behaviour: an exploration study based on grounded theory. Chin. J. Manag. 17, 1823–1830. doi: 10.3969/j.issn.1672-884x.2020.12.010

[ref46] Sun H. Zhang P Xiao X. (2007). “A research model of relationship quality in e-commerce: connecting IS factors with marketing profitability.” in *Proceedings of the 13th Americas Conference on Informaiton Systems*; August 2007; Key stone: AMCIS, 290–305.

[ref47] Thomson M. (2006). Human brands: investigating antecedents to consumers' strong attachments to celebrities. J. Mark. 70, 104–119. doi: 10.1509/jmkg.70.3.104

[ref48] Thomson M. Johnson A. R. (2006). Marketplace and personal space: investigating the differential effects of attachment style across relationship contexts. Psychol. Mark. 23, 711–726. doi: 10.1002/mar.20125

[ref49] Thomson M. Macinnis D. J. Park C. W. (2005). The ties that bind: measuring the strength of consumers' emotional attachments to brands. J. Consum. Psychol. 15, 77–91. doi: 10.1207/s15327663jcp1501_10

[ref50] Torkzadeh G. Koufteros X. Pflughoeft K. (2003). Confirmatory analysis of computer self-efficacy. Struct. Equ. Model. 10, 263–275. doi: 10.1207/S15328007SEM1002_6

[ref52] Vlachos P. A. Theotokis A. Pramatari K. (2010). Consumer-retailer emotional attachment: some antecedents and the moderating role of attachment anxiety. Eur. J. Mark. 44, 1478–1499. doi: 10.1108/03090561011062934

[ref53] Wang F. Head M. (2007). How can the web help build customer relationships? An empirical study on E-tailing. Inf. Manag. 44, 115–129. doi: 10.1016/j.im.2006.10.008

[ref54] Xie X. Z. Zhu Y. Y. (2019). Analysis of the problems behind the popularity of short videos. Publish. Sci. 27, 86–91. doi: 10.13363/j.publishingjournal.2019.01.028

[ref55] Yang M. S. Hu S. G. Kpandika B. E. Liu L. (2021b). Effects of social attachment on social media continuous usage intention: the mediating role of affective commitment. Hum. Syst. Manag. 40, 619–631. doi: 10.3233/HSM-201057

[ref56] Yang X. X. Li B. Q. (2019). On the communication mechanism of China-related content in international social media platforms. Acad. J. Zhongzhou 9, 162–167. doi: 10.3969/j.issn.1003-0751.2019.09.026

[ref57] Yang M. S. Zhang W. S. Ruangkanjanases A. Zhang Y. (2021a). Understanding the mechanism of social attachment role in social media: a qualitative analysis. Front. Psychol. 12:720880. doi: 10.3389/fpsyg.2021.720880, PMID: 34421773 PMC8378210

[ref58] Zhao X. L. Jie M. (2020). Analysis on the interaction characteristics of users and contents of instant video based on the TikTok application-focusing on the case of Chinese men in their twenties. J. Korean Soc. Des. Cult. 26, 527–537. doi: 10.18208/ksdc.2020.26.2.527

